# A transcription factor that promotes proliferation, migration, invasion, and epithelial–mesenchymal transition of ovarian cancer cells and its possible mechanisms

**DOI:** 10.1186/s12938-021-00919-y

**Published:** 2021-08-16

**Authors:** Yingying Qi, Kexin Mo, Ting Zhang

**Affiliations:** Department of Gynecology, the Fifth Affiliated Hospital of Guangzhou Medical University, No. 621 Harbor Road, Guangzhou, 510700 Guangdong China

**Keywords:** Ovarian cancer, Non-SMC condensin I complex subunit H, GATA binding protein 3, Migration, Invasion

## Abstract

**Background:**

Ovarian cancer is one of the most common gynecological malignancies with the high morbidity and mortality. This study was aimed to explore the role of non-structure maintenance of chromosomes condensin I complex subunit H (NCAPH) in the progression of ovarian cancer (OC) and the transcription regulatory effects of GATA binding protein 3 (GATA3) on this gene.

**Methods:**

Firstly, NCAPH and GATA3 expression in OC tissues and several human OC cell lines was, respectively, evaluated by TNMplot database and Western blot analysis. Then, NCAPH was silenced to assess the proliferation, migration, and invasion of OC cells in turn using CCK-8, wound healing, and transwell assays. Western blotting was used to determine the expression of epithelial--mesenchymal transition (EMT)-related proteins and PI3K/PDK1/AKT signaling proteins. The potential binding sites of GATA3 on NCAPH promoter were predicated using JASPAR database, which were verified by luciferase reporter assay and chromosomal immunoprecipitation. Subsequently, GATA3 was overexpressed to examine the biological functions of OC cells with NCAPH silencing.

**Results:**

NCAPH and GATA3 expression was significantly upregulated in OC tissues and cell lines. NCAPH loss-of-function notably inhibited the proliferation, migration, invasion, and EMT of OC cells. Moreover, the expression of p-PI3K, PDK1, and p-AKT was downregulated after NCAPH knockdown. Furthermore, GATA3 was confirmed to bind to NCAPH promoter. GATA3 overexpression alleviated the inhibitory effects of NCAPH silencing on the proliferation, migration, invasion, EMT, and expression of proteins in PI3K/PDK1/AKT pathway of OC cells.

**Conclusion:**

To sum up, NCAPH expression transcriptional activation by GATA3 accelerates the progression of OC via upregulating PI3K/PDK1/AKT pathway.

## Introduction

Ovarian cancer (OC) is one of the deadliest gynecological malignancies, with high incidence and mortality rates worldwide, severely compromising women’s health and quality of life [1, 2]. Radical surgery and chemotherapy are considered as two important methods for treating OC, but these methods do not appear to reduce the high recurrence rate of OC or improve the prognosis of the patient with OC [3]. The 5-year survival rate of patients with OC globally remains < 35% [4]. Thus, it is urgent to obtain an improved understanding regarding the pathogenesis underlying OC to identify and develop more effective therapeutic targets for the treatment of this disease.

Non-structure maintenance of chromosomes condensin I complex subunit H (NCAPH) is a member of the condensin I complex, which belongs to a recently defined superfamily of proteins termed kleisins [5]. Substantial evidence exists to suggest that abnormal NCAPH expression is involved in the pathogenesis of multiple human malignancies. For instances, NCAPH is significantly upregulated in prostate cancer, and NCAPH silencing suppresses cell proliferation and metastasis [6, 7]. Compelling evidence indicates that NCAPH functions as an oncogene in endometrial cancer and breast cancer, and its highly expression represents a poor prognosis [8, 9]. Zhan et al firstly demonstrated that NCAPH is upregulated in serous ovarian cancer, which might be involved in the carcinogenesis of this disease and carboplatin resistance, providing a potential prognostic indicator for patients with carboplatin resistance in serous ovarian cancer [10]. However, the role of NCAPH in the progression of OC remains to be elucidated.

GATA binding protein 3 (GATA3), one of the GATA family, is a highly conserved zinc-finger transcription factor which plays a crucial role in diverse cellular processes, including proliferation, invasion, DNA repair, and senescence [11–13]. The association of GATA3 with human cancers has been reported in a large amount of human cancers, such as liver cancer, breast cancers, and endometrial carcinomas [14–16]. In high-grade serous ovarian cancer, GATA3 acts as an oncogenic protein and high expression of GATA3 is associated with poor prognosis in patients [17]. The present study investigated whether GATA3 can transcriptionally activate NCAPH expression to participate in the progression of OC.

In this study, we aimed to explore the role of GATA3 and NCAPH in the progression of OC. NCAPH and GATA3 expression in OC tissues was evaluated using TNMplot database (https://www.tnmplot.com/). After detection of NCAPH and GATA3 levels in several OC cell lines, the effects of NCAPH on proliferation, migration, invasion, and epithelial--mesenchymal transition (EMT) of OSCC cells as well as the regulatory effects and potential mechanisms of GATA3 on NCAPH in OC progression were explored. Our findings might provide a possible new direction for OC treatment.

## Results

### NCAPH is highly expressed in OC tissues and cell lines

**Fig. 1:**
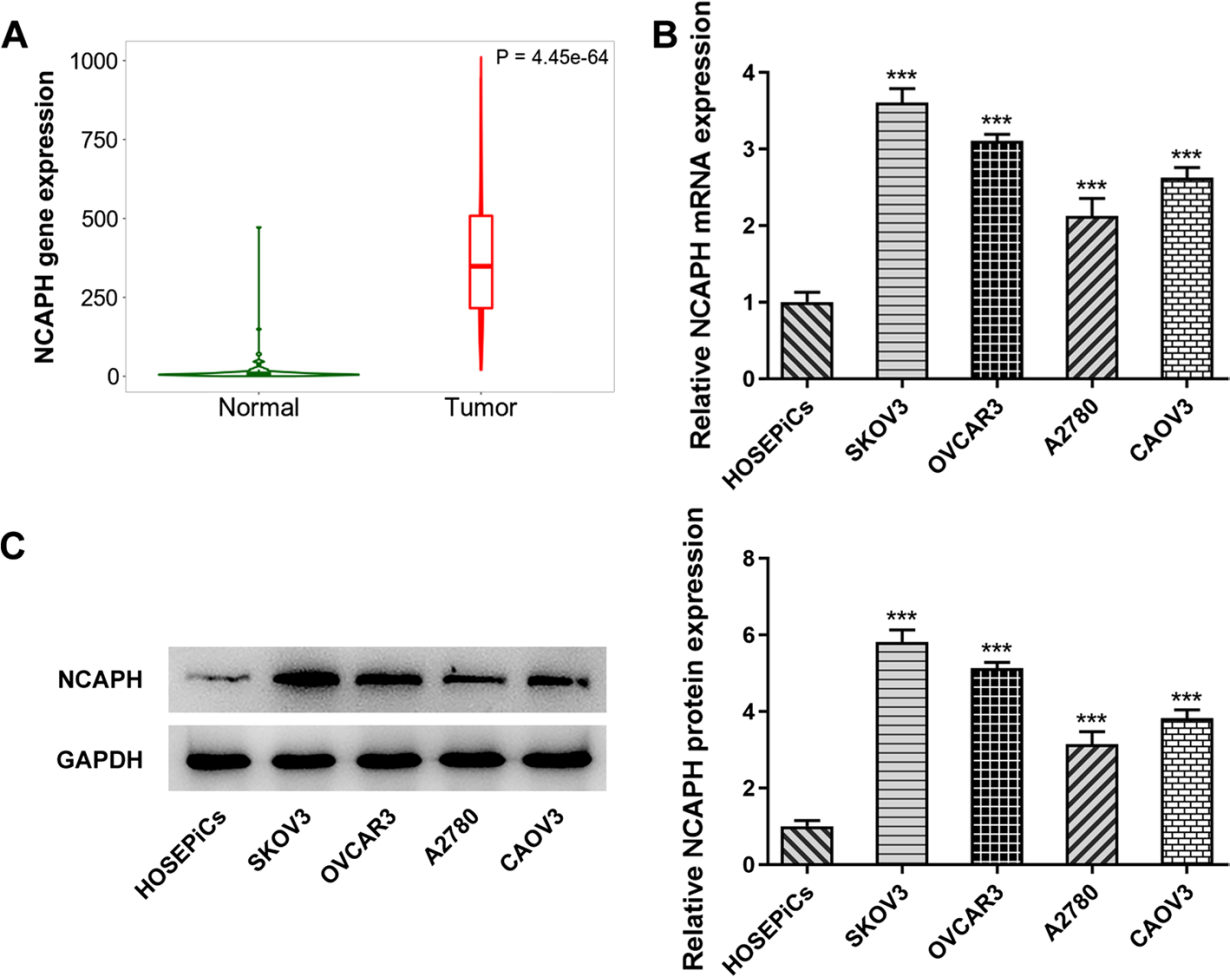
NCAPH was highly expressed in OC tissues and cell lines. **A** The level of NCAPH was assessed using TNMplot (https://www.tnmplot.com/). **B**, **C** NCAPH expression in several OC cell lines (SKOV3, OVCAR3, A2780, and CAOV3) and one human ovarian epithelial cell line HOSEpiC was tested by RT-qPCR and Western blot analysis. All experiments were performed in triplicates. ^***^P < 0.001 vs. HOSEpiC

To investigate the role of NCAPH in the progression of OC, the expression of this gene in OC tissues was analyzed with TNMplot database. As shown in Fig. 1A, NCAPH expression was significantly upregulated in the tumor group compared with the normal group. Then, notably elevated expression of NCAPH was observed in the OC cell lines (SKOV3, OVCAR3, A2780, and CAOV3) as compared to the HOSEpiC group and SKOV3 cell line was selected to perform the subsequent experiments due to its lowest NCAPH expression level (Fig. 1B, C). These results suggest that NCAPH is overexpressed in OC tissues and cell lines.

### Depletion of NCAPH inhibits proliferation, migration, invasion, and EMT of OC cells

**Fig. 2:**
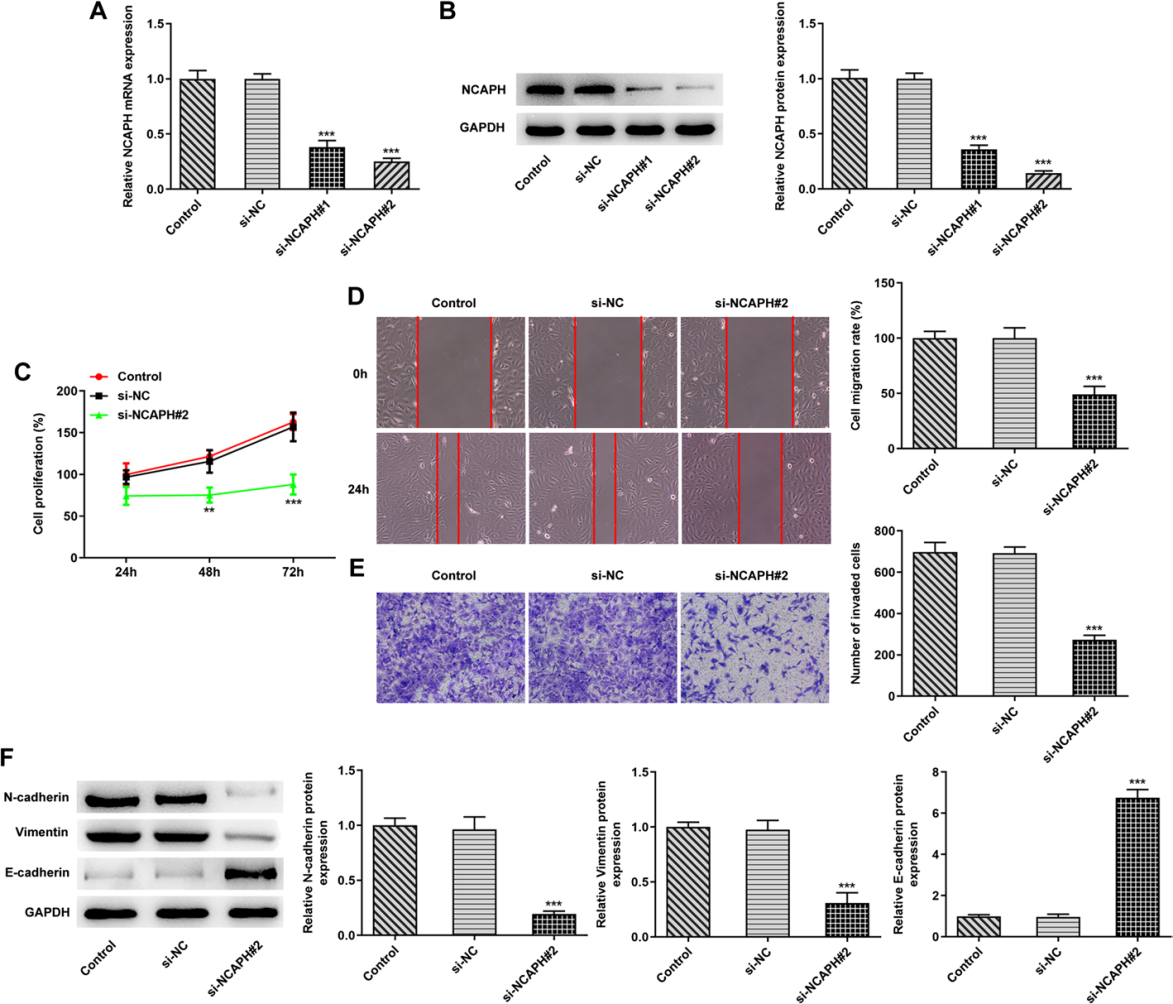
Depletion of NCAPH suppressed proliferation, migration, invasion, and EMT of OC cells. **A**, **B** NCAPH expression in SKOV3 cells was determined by means of RT-qPCR and Western blot analysis after transfection. **C** Cell viability was measured with CCK-8 assay. **D** Cell migration was tested using wound healing assay. **E** The invasive ability of SKOV3 cells was examined with transwell assay. **F** Western blotting was employed to evaluate the expression of EMT-related proteins. Data were obtained from three independent experiments. ^**^P < 0.01, ^***^P < 0.001 vs. si-NC

Then, with the objective of identifying the impact of NCAPH on the progression of malignant behaviors of OC, NCAPH was silenced by transfection with siRNAs. As what is observable from Fig. 2A, B, NCAPH expression was remarkably downregulated in the SKOV3 cells transfected with si-NCAPH#1 or si-NCAPH#2 relative to the si-NC group, and si-NCAPH#2 was chosen for the following transfection due to its better knockdown efficiency. Significantly, results shown in Fig. 2C revealed that NCAPH silencing inhibited cell viability when compared to the si-NC group. Additionally, the abilities of SKOV3 cell migration and invasion were markedly restrained in the NCAPH silenced group (Fig. 2D, E). Concurrently, NCAPH knockdown downregulated N-cadherin and Vimentin expression, accompanied by upregulated E-cadherin expression as compared to the si-NC group (Fig. 2F). These data imply the inhibitory effects of NCAPH silencing on the progression of OC.

### Depletion of NCAPH inactivated the phosphatidylinositol 3-kinase (PI3K)/3-phosphoinositide-dependent protein kinase 1 (PDK1)/AKT signaling pathway in OC cells

**Fig. 3:**
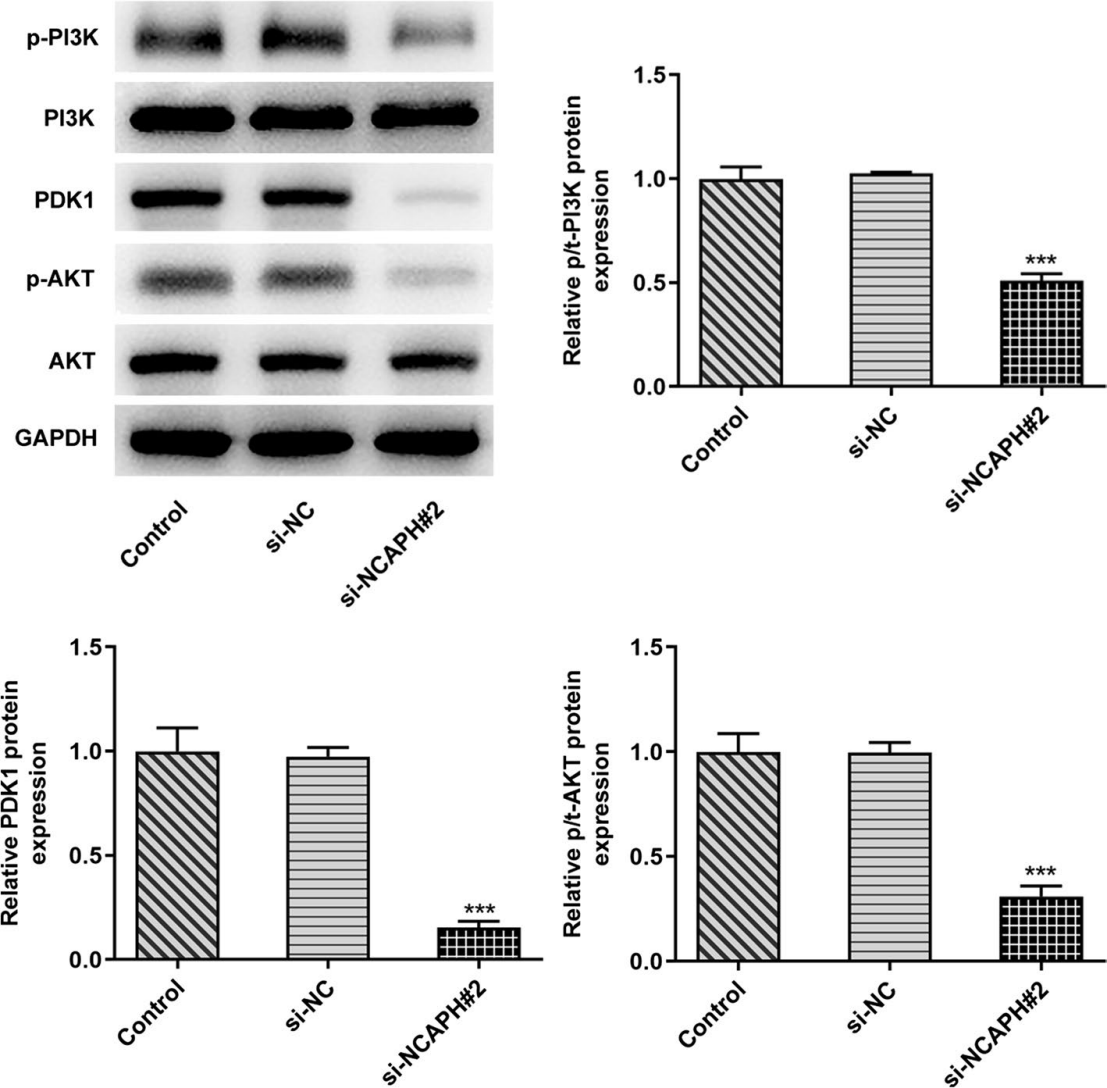
NCAPH knockdown inactivated the PI3K/AKT signaling pathway in OC cells. The expression of p-PI3K, PDK1, and p-AKT in SKOV3 cells after NCAPH silencing was detected using Western blot assay. All experiments were performed in triplicates. ^***^P < 0.001 vs. si-NC

To explore the downstream signaling regulated by NCAPH, expression of proteins in PI3K/AKT signaling pathway was examined with Western blot analysis. It could be found that NCAPH silencing conspicuously reduced phosphor (p)-PI3K, PDK1, and p-AKT expression levels when compared to the si-NC group (Fig. 3). These data provide evidence that NCAPH downregulation can suppress the PI3K/PDK1/AKT signaling pathway to attenuate the OC progression.

### GATA3 transcriptionally activates NCAPH expression in OC cells by binding to the NCAPH promoter

**Fig. 4:**
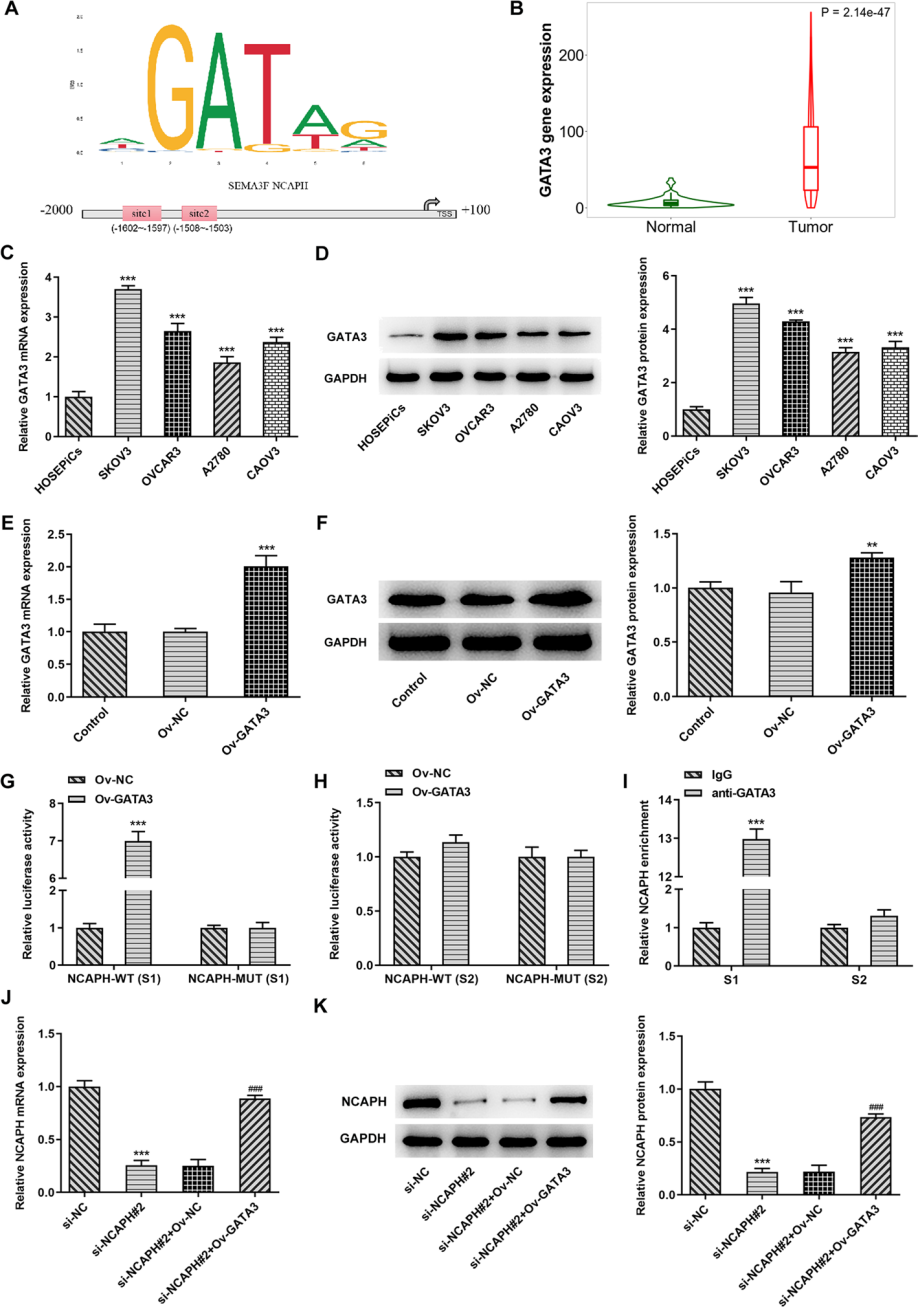
GATA3 activated NCAPH expression in OC cells by binding to the NCAPH promoter. **A** GATA3 binding with the promoter of NCAPH was predicated by JASPAR databases. **B** NCAPH level in OC tissues was tested by TNMplot (https://www.tnmplot.com/). **C**, **D** NCAPH expression in several OC cell lines (SKOV3, OVCAR3, A2780, and CAOV3) and one human ovarian epithelial cell line HOSEpiC was evaluated with RT-qPCR and Western blot analysis. ^***^P < 0.001 vs. HOSEpiC. (**E**, **F**) GATA3 expression was measured using RT-qPCR and Western blotting after GATA3 overexpression. Data were obtained from three independent experiments. ^**^P < 0.01, ^***^P < 0.001 vs. Ov-NC. **G**, **H** The binding between GATA6 and the promoter regions (S1 and S2) was determined by luciferase reporter assays. Results were generated from three independent experiments. ^***^P < 0.001 vs. Ov-NC. **I** Chromosomal immunoprecipitation assay was used to detect the direct binding of GATA3 to NCAPH promoter. All experiments were performed in triplicates. ^***^P < 0.001 vs. IgG. **J**, **K** NCAPH levels were assessed by RT-qPCR and Western blot analysis. ^***^P < 0.001 vs. si-NC; ^###^*P* < 0.001 vs. si-NCAPH#2+Ov-NC

To further clarify the molecular mechanism of NCAPH in the malignant behaviors of OC, GATA3 was predicted as a potential transcription factor in NCAPH upstream using the JASPAR database (http://jaspar.genereg.net/). Two possible binding sites, site1 (S1): − 1062 to − 1597 and site2 (S2): − 1508 to − 1503, of GATA3 to NCAPH promoter are presented in Fig. 4A. Then, GATA3 expression in OC tissues and different OC cell lines was examined. Notably upregulated GATA3 expression was observed in both OC tissues and cell lines and the highest-level GATA3 was found in the SKOV3 cells (Fig. 4B–D). Moreover, GATA3 was overexpressed by transfection with pcDNA3.1 plasmid, and the successful overexpressing effects are found in Fig. 4E, F. The subsequent dual luciferase reporter assay indicated that luciferase reporter plasmid with NCAPH-WT (S1) was activated by GATA3 overexpression, whereas there was no significant difference in the NCAPH-WT (S2) in the Ov-GATA3 and OV-NC groups (Fig. 4G, H). Furthermore, results of ChIP assays exhibited in Fig. 4I revealed that notable enrichment of NCAPH promoter sequence S1 was obtained through immunoprecipitation with an anti-GATA3 antibody, but not with the control IgG antibody. However, the enrichment of NCAPH promoter sequence S2 presented no significance in the anti-GATA3 group. Besides, gain function of GATA3 remarkably enhanced NCAPH in SKOV3 cells with NCAPH silencing compared with the si-NCAPH#2+Ov-NC group (Fig. 4J, K). Through the above findings we proved that GATA3 transcriptionally activates NCAPH expression in OC cells by binding to the NCAPH promoter.

### GATA3 overexpression cripples the inhibitory effects of NCAPH silencing on the progression of OC

**Fig. 5:**
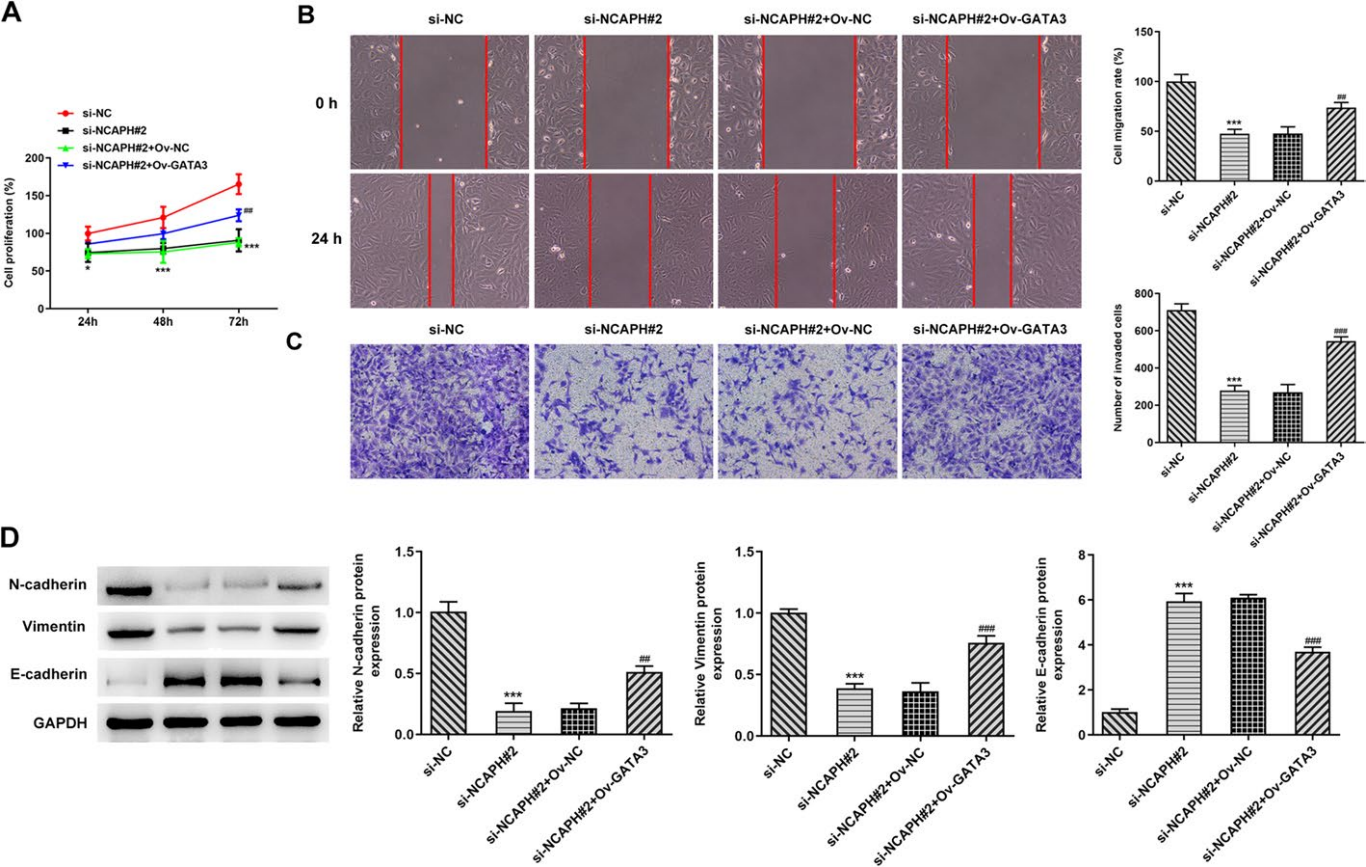
GATA3 overexpression alleviated the inhibitory effects of NCAPH silencing on the progression of OC. **A** Cell viability was tested using CCK-8 assay. **B** Cell migration was assessed by wound healing assay. **C** Cell invasion was evaluated by means of transwell assay. **D** The expression of EMT-related proteins was determined using Western blot analysis. Results were generated from three independent experiments. ^*^P < 0.05, ^***^P < 0.001 vs. si-NC; ^##^*P* < 0.01, ^###^*P* < 0.001 vs. si-NCAPH#2+Ov-NC

The malignant behaviors of OC were analyzed after GATA3 overexpression in SKOV3 cells with NCAPH silencing. It was found that gain function of GATA3 conspicuously restored the inhibitory effects of NCAPH knockdown on the proliferation, migration, and invasion of SKOV3 cells when compared to the si-NCAPH#2+Ov-NC group (Fig. 5A–C). Additionally, the expression of N-cadherin and Vimentin was elevated while that of E-cadherin was reduced in the si-NCAPH#2+Ov-GATA3 group relative to the si-NCAPH#2+Ov-NC group (Fig. 5D). These observations suggest that GATA3-induced NCAPH upregulation regulates the progression of OC.

### GATA3 overexpression abrogates the impact of NCAPH silencing on the PI3K/ PDK1/AKT signaling pathway

**Fig. 6:**
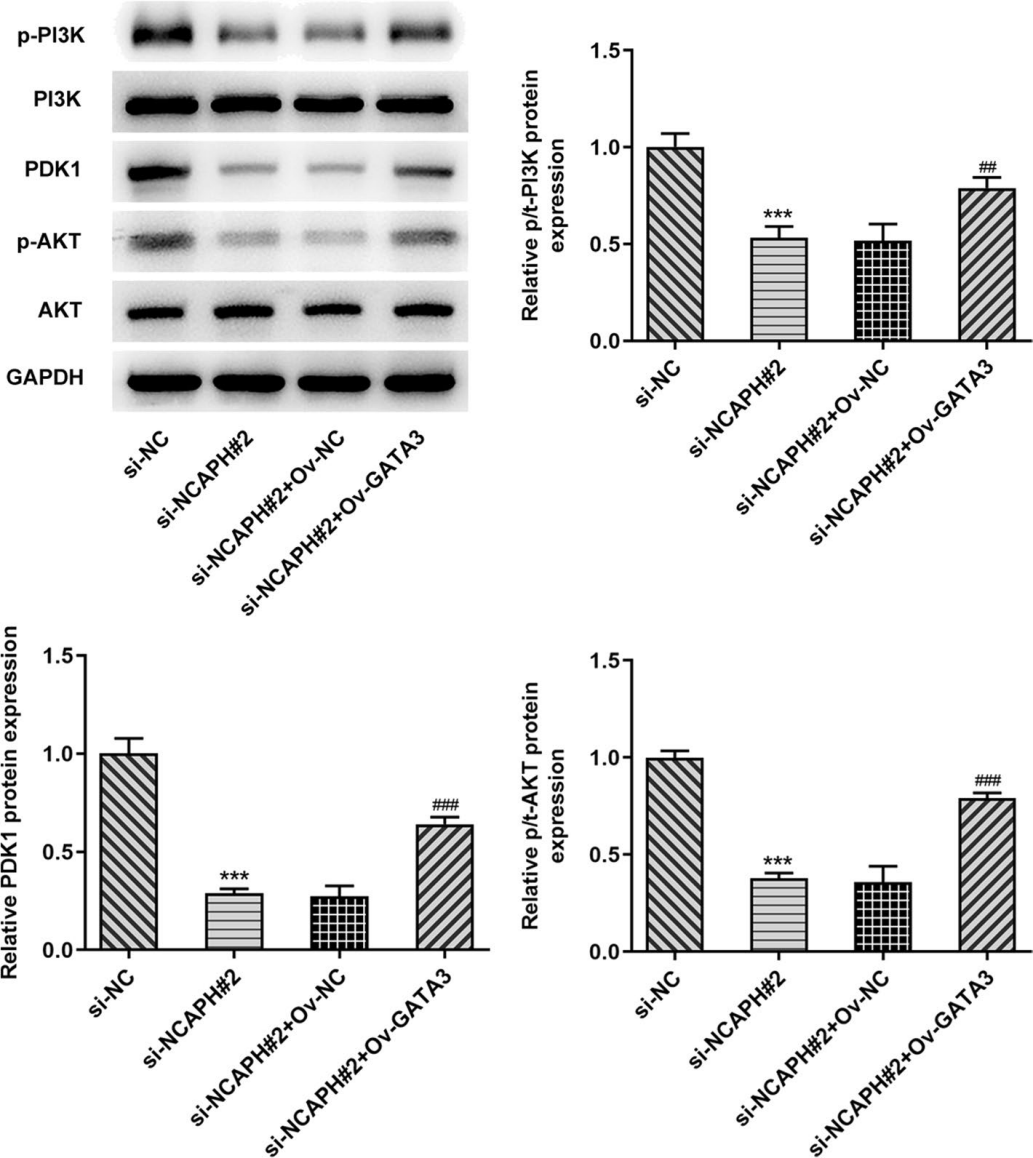
GATA3 overexpression abrogated the impact of NCAPH deletion on the PI3K/AKT signaling pathway. The expression of p-PI3K, PDK1, and p-AKT in SKOV3 cells after NCAPH knockdown was determined with Western blot analysis. All experiments were performed in triplicates. ^***^P < 0.001 vs. si-NC; ^##^*P* < 0.01, ^###^*P* < 0.001 vs. si-NCAPH#2+Ov-NC

Finally, the expression of proteins in PI3K/PDK1/AKT signaling pathway was evaluated with Western blot analysis. As displayed in Fig. 6, gain function of GATA3 partially counteracted the diminished effects of NCAPH deletion on the expression of p-PI3K, PDK1, and p-AKT in SKOV3 cells, suggesting that GATA3-induced NCAPH activation regulates the progression of OC via affecting PI3K/PDK1/AKT signaling pathway.

## Discussion

The present study identified that NCAPH is notably elevated in OC samples and cell lines. Deletion of NCAPH inhibits the proliferation, migration, invasion, and EMT of OC cells, which is reversed by the upregulation of GATA3 transcription factor, suggesting GATA3-induced NCAPH promotes OC progression.

An increasing number of researches have validated the importance of the condensin I complex, a structural component of chromosomes during mitosis, in the tumorigenesis [8, 18, 19]. NCAPH is one of the three important members of non-SMC subunits in condensin I complex, the other two are NCAPG and NCAPD2, which plays vital roles in maintaining the stability of chromosome organization to ensure the chromosome-wide gene regulation in mitotic and meiotic cell cycles [20]. NCAPH has been demonstrated to be elevated in a large amount of human cancers. In endometrial cancer, significantly upregulated NCAPH expression is observed in clinical tissues when compared to the normal tissues, which is associated with poor clinicopathologic characteristics [9]. Another study has determined that aberrantly high NCAPH overexpression is required for proliferation, migration, and invasion of non-small cell lung cancer [21]. A recent study performed by Yin et al suggested that NCAPH expression is upregulated in colon cancerous cell lines and tissues and loss function of NCAPH restrains proliferation and migration of colon cancerous cells [22]. So far, only one previous research has revealed the high expression of NCAPH in OC by analyzing the microarray datasets GDS1381 and GDS3592 [10]. This study is the first to explore the function of NCAPH in the malignant behaviors of OC. We demonstrated that NCAPH was upregulated in OC tissues using TNMplot database, which was in agreement with the aforementioned microarray datasets. Importantly, deletion of NCAPH conspicuously inhibited the proliferation, migration, invasion, and EMT of OC cells.

Previous research has revealed that PI3K/AKT signaling is a well-known driver of tumorigenesis, and activation of this pathway is closely associated with cancer migration, invasion, and EMT, making it accountable for the high aggressiveness of cancers [23–25]. PDK1 is one of the main components of the PI3K/AKT pathway. When PI3K is activated, the signal is transmitted to PDK1, and then the downstream AKT signaling pathway is activated by PDK1 [23]. Research has proposed that inhibition of the PI3K/AKT signaling pathway can play antitumor effect in a huge number of human cancers, such as leukemia, breast cancer, bladder cancer, and ovarian cancer [26–29]. It is worthy of note that NCAPH can regulate the PI3K/AKT signaling pathway in cervical cancer [30]. The present study demonstrated that NCAPH silencing inactivated the PI3K/PDK1/AKT signaling.

According to JASPAR database analyses, GATA3 could potentially bind to the promoter of NCAPH. GATA3 is a member of the zinc-finger transcription factor family, whose members contain zinc-finger DNA binding domains that bind to consensus 5′-(A/T) GATA (A/G)-3′ motifs [11, 31]. The tumor inhibiting or tumor promoting effects of GATA3 have been widely reported in different malignant tumors. For instance, loss of GATA3 promotes the proliferation, migration, invasion, and EMT of bladder cancer, gastric cancer, breast cancer, and osteosarcoma [32–35]. On the contrary, Peng et al suggested that elevated GATA3 expression is associated with enhanced self-renewal and proliferation of neuroblastoma cells [36]. GATA3 is overexpressed in neck squamous cell carcinoma, which promotes tumor invasiveness *in vitro* and *in vivo* [13]. GATA3 expression is associated with an aggressive phenotype and poor patient survival in endometrial carcinomas [16]. Importantly, GATA3 acts as an oncogenic protein and high expression of GATA3 is associated with poor prognosis in patients with high-grade serous ovarian cancer [17]. In a follow-up study performed in USA, GATA3 enhances the stemness of tumor cells and promotes the proliferation and migration of serous ovarian cancer cells [37]. In the present study, remarkably upregulated GATA3 expression was observed in OC tissues and cell lines. The subsequent experiments demonstrated that GATA3 could transcriptionally activate NCAPH expression in OC cells by binding to the NCAPH promoter. Further analysis suggested that GATA3 overexpression crippled the inhibitory effects of NCAPH silencing on the progression of OC. Additionally, NCAPH deletion induced decrease in the expression of proteins in PI3K/PDK1/AKT signaling was notably upregulated after GATA3 upregulation, suggesting that GATA3-induced NCAPH activation regulates the progression of OC via affecting PI3K/PDK1/AKT signaling pathway.

## Conclusion

To conclude, the present study is the first to demonstrate that NCAPH silencing inhibits the proliferation, migration, invasion, and EMT of OC cells. GATA3 is determined to have a direct regulatory effect on the transcription of NCAPH by binding to NCAPH promoter. Mechanically, GATA3-induced NCAPH activation regulates the progression of OC via affecting PI3K/PDK1/AKT signaling pathway. These findings shed light on the mechanism of GATA3/NCAPH in OC and provides possible targets for OC treatment. The lack of data about the effects of NCAPH and GATA3 on the growth and metastasis of OC *in vivo* as well as the usage of agonists or inhibitors of pathway PI3K/PDK1/AKT signaling are limitations of the present study, which will be investigated in the next experiments to further support the conclusions in this study.

## Materials and methods

### Cell culture

Four human OC cell lines (SKOV3, OVCAR3, A2780, and CAOV3) and one human ovarian epithelial cell line HOSEpiC were obtained from Chinese Academy of Sciences Cell Bank (Shanghai, China). Cells were maintained in RPMI1640 medium (Gibco, Grand Island, USA) containing 10% fetal bovine serum (FBS, Gibco, Grand Island, USA). The incubator was set as 5% CO_2_ humidified atmosphere at 37 °C.

### Cell transfection

SKOV3 cells in logarithmic phase were inoculated in a 96-well plate and cultured until 70% confluence. Small interfering RNA (siRNA) targeting NCAPH (si-NCAPH#1 and si-NCAPH#2) and the negative control (si-NC), GATA3 overexpression pcDNA3.1 plasmid (Ov-GATA3), and the empty vector plasmid (Ov-NC) were provided by Shanghai GenePharma Co., Ltd. The transfection was done using Lipofectamine 3000 transfection reagent (Invitrogen, Madison, CA, USA) as provided by the manufacturer. After transfection for 48 h, the transfection efficiency was evaluated using reverse transcription quantitative PCR (RT-qPCR) and Western blot analysis.

### Cell viability assay

After transfection, SKOV3 cells were seeded at 5 × 10^3^ cells per well in 96-well plates. Cell Counting Kit-8 (CCK-8; Shanghai Yeasen Biotechnology Co., Ltd.) was used for the detection of cell viability. At 24, 48, and 72 h after the cells were seeded, 10 μl of the CCK-8 solution was added into each well and cells were incubated at 37 °C for 2 h. The optical density was measured at 450 nm with a microplate reader (Bio-Rad Laboratories, Inc.).

### Wound healing assay

The ability of cell migration was determined using wound scratch assay. Briefly, SKOV3 cells (3 × 10^6^ cells/well) were plated into a 6-well plate. When cells reached a subconfluency of 90 %, serum-free RPMI1640 medium was utilized to incubate cells overnight prior. Then, a sterilized 100-μl pipette tip was utilized to create a wound on the cellular surface. After incubation for 24 h, the debris was eluted by PBS. Images were obtained at 0 h and 24 h under a light microscope (Olympus, Tokyo, Japan). Quantitative analysis of the wound healing area was performed using Image J software (version 1.52r; National Institutes of Health).

### Transwell assay

A transwell chamber invasion assay with 8-μm pore inserts coated with Matrigel (BD Biosciences) was employed to examine the invasive ability of SKOV3 cells. Cells (2.5 × 10^4^) resuspended in 200-μl serum-free medium were seeded into the upper chamber. In the lower chamber, 600-μl RPMI1640 medium supplemented with 10% FBS was added as a chemoattractant. The plate was incubated for 48 h at 37 °C in an incubator with 5% CO_2_. The invaded cells were fixed in 4% paraformaldehyde for 20 min at 37 °C, and stained with 1% crystal violet for 30 min. The number of cells that migrated to the lower side was counted using an inverted light microscope (Olympus Corporation) from five randomly selected fields.

### Dual luciferase reporter assay

Luciferase reporter plasmids (Promega Corporation) were constructed with wild-type (WT) and mutant-type (MUT) 3′-untranslated regions of NCAPH promoter. The luciferase reporter plasmids and GATA3-expressing plasmid were co-transfected into SKOV3 cells using Lipofectamine^®^ 3000 reagent (Invitrogen; Thermo Fisher Scientific, Inc.). Luciferase activities were measured 48 h after transfection using the Dual Luciferase Reporter Assay Kit (Promega, Madison, WI, USA).

### Chromatin immunoprecipitation (ChIP) assay

The binding of GATA3 to the NCAPH promoter was examined using a ChIP assay with a kit (Beyotime Institute of Biotechnology) as per the manufacturer’s suggestions. SKOV3 cells were cross-linked by 1% formaldehyde for 15 min at room temperature, and the cell lysates in the lysis buffer were sonicated to achieve chromatin fragments. ChIP was conducted after incubation with anti-GATA3 antibody (Cell Signaling Technology, Danvers, MA, USA). The enrichment of indicated proteins in NCAPH promoter was evaluated by PCR.

### RT-qPCR

Total RNA was isolated from OC cells using TRIzol reagent ((Invitrogen, Carlsbad, CA, USA)). Then, a SuperScript IV First-Strand Synthesis system (Invitrogen; Thermo Fisher Scientific, Inc.) was used to synthesize the complementary DNA following manufacturer’s recommendations. The temperature protocol for this step was as follows: 70 °C for 5 min, 37 °C for 5 min, and 42 °C for 1 h. PCR was performed using SYBR Green PCR Master Mix (Applied Biosystems) on an ABI 7300 thermal-recycler (Applied Biosystems; Thermo Fisher Scientific, Inc.). The following thermocycling conditions were used: Initial denaturation at 95 °C for 10 min; followed by 40 cycles of denaturation at 95 °C for 15 sec and annealing at 60 °C for 1 min; and a final extension of 10 min at 72 °C. The relative mRNA expression was calculated using the 2^−ΔΔCq^ method.

### Western blot analysis

Total cell proteins were extracted from lysed cells with RIPA lysis buffer (Beyotime). The protein concentration was tested with BCA method (Beyotime). A total of 40 µg of protein was separated by 10% SDS-polyacrylamide gels, which was then transferred onto nitrocellulose membranes (Merck KGaA). After blocking with 5% skimmed milk for 1.5 h, the membranes were first probed with specific primary antibodies at 4 °C overnight. Subsequently, these blots were incubated with peroxidase-conjugated secondary antibody for 1.5 h at room temperature. The proteins were finally visualized under enhanced chemiluminescence (EMD Millipore). Band densities of target proteins were normalized to that of GAPDH and quantified using Image J software.

### Statistical analysis

Quantitative data are presented as the means ± standard deviation of three independent experiments. The monitoring data were analyzed using GraphPad Prism 8.0 (GraphPad Software, Inc.). ANOVA with Tukey’s post hoc test was employed to analyze significance among multiple groups. *P* < 0.05 was considered to indicate a statistically significant difference.

## Data Availability

The datasets used and/or analyzed during the present study are available from the corresponding author on reasonable request.
